# The Psychology of Sustainability and Sustainable Development for Well-Being in Organizations

**DOI:** 10.3389/fpsyg.2017.01534

**Published:** 2017-09-19

**Authors:** Annamaria Di Fabio

**Affiliations:** Department of Education and Psychology, Psychology Section, University of Florence Florence, Italy

**Keywords:** psychology of sustainability, sustainable development, well-being, sustainable organizations, primary prevention perspective in organizations

## Abstract

This article discusses the contribution of the psychology of sustainability and sustainable development to well-being in organizations from a primary prevention perspective. It deals with sustainability not only in terms of the ecological, economic, and social environment but also in terms of improving the quality of life of every human being. The psychology of sustainability and sustainable development is seen as a primary prevention perspective that can foster well-being in organizations at all the different levels going from the worker, to the group, to the organization, and also to inter-organizational processes. The possibilities for further research and interventions are also discussed.

## Introduction

Just as the 21st century as a whole is characterized by complexity (Landy and Conte, 2016), acceleration (Rosa, 2013), change (Weiten et al., 2014), and globalization (Savickas, 2011; Blustein, 2013; Guichard, 2013), so the labor market is characterized by insecurity, economic instability, and ongoing turbulence (Blustein, 2011; Savickas, 2011). In this scenario, the well-being of individuals and organizations is particularly at risk (Van den Heuvel et al., 2010; Di Fabio, 2014b; Di Fabio and Kenny, 2016). Opportunities are accordingly arising for a new area of research and intervention, namely the psychology of sustainability (Di Fabio, 2016a, 2017) in terms of sustainable development for well-being in organizations (Tetrick and Peiró, 2012; Di Fabio, 2016a, 2017; Peiró, 2017).

The United Nations has proposed 17 sustainable development goals: no poverty; no hunger; good health and well-being; quality education; gender equality; clean water and sanitation; affordable and clean energy; decent work and economic growth; industry, innovation, and infrastructure growth; reduction in inequality; sustainable cities and communities; responsible consumption and production; climate action; life below water; life on land; peace, justice, and strong institutions; partnerships to achieve the goals (United Nations, 2015). These goals underline the importance of increasing opportunities for progress and promoting the development of individuals, families, and communities to ensure sustainable development and global growth. This is particularly true also for organizations (Di Fabio, 2017). Well-being is a key sustainable development goal and a fundamental requirement for good health, which is defined as “a state of complete physical, mental, spiritual, and social well-being and not merely the absence of disease or infirmity” (World Health Organization, 1998, 2007; Macik-Frey et al., 2007). Well-being is therefore an essential part of organizational life and human resources management (De Smet et al., 2007; Di Fabio, 2017).

This extended definition of health sees healthy people as flourishing and resilient workers and emphasizes the importance of a positive work environment in promoting employee health, well-being, and performance, particularly from an organizational positive psychology perspective (Di Fabio, 2014b; Snyder et al., 2014; Di Fabio and Gori, 2016a,b; Di Fabio and Kenny, 2016).

## Well-Being and the Challenge of the Primary Prevention Approach in Organizations

Tetrick and Peiró (2012) discuss the passage from ill health to positive health in organizations, as well as the promotion of health, well-being, and flourishing (Hofmann and Tetrick, 2003; Schaufeli, 2004; Macik-Frey et al., 2007). This involves focusing on people’s talents and gifts to achieve high performance, satisfaction, and well-being (Quick, 1999). The 21st century has seen the introduction of a positive psychology approach to well-being based on the enhancement of individual and organizational resources (Di Fabio, 2014b, 2017; Di Fabio and Kenny, 2016) to help deal with the complexity of the post-modern era and to sustain the well-being of people, groups, and organizations. This positive psychology approach (Seligman and Csikszentmihalyi, 2000; Seligman, 2002) highlights study success and excellence – rather than anything negative – to promote well-being at the individual, group, organization, and inter-organization level (Henry, 2005). A call is made for cross-level interactions where individual approaches are complemented by collective approaches, combining short-term with long-term goals and outcomes (Hofmann and Tetrick, 2003), and for the introduction of a proactive and anticipatory approach to enhance particularly a primary prevention perspective (Peiró, 2008; Tetrick and Peiró, 2012; Di Fabio and Kenny, 2015, 2016). A shift has occurred from the traditional focus on the elimination of risks to employees’ safety and health (Quick and Tetrick, 2003) to a more recent focus on the promotion of growth and positive experiences (Kelloway et al., 2008) with the emphasis on the development of a safe and healthy work environment (Tetrick and Peiró, 2012).

From a primary prevention perspective (Hage et al., 2007; Kenny and Hage, 2009; Di Fabio and Saklofske, 2014b; Di Fabio and Kenny, 2015), increasing the resources of individuals is crucial to building strength (Seligman, 2002; Di Fabio and Palazzeschi, 2012, 2015; Di Fabio et al., 2014, 2016a; Di Fabio, 2015). Primary prevention is aimed at preventing the development of a problem before it starts and, at the same time, promoting psychological well-being. This is achieved by building on the resources and strengths of workers in a positive prevention framework (Di Fabio, 2016a) and can be referred to as positive organizational health psychology (Di Fabio, 2017). Here the focus is on promoting – with interventions at different levels – individual, group, organizational, and inter-organizational health (Henry, 2005; Di Fabio, 2017). The psychology of sustainability (Di Fabio, 2017) and sustainable development can be seen therefore as a new approach to promoting well-being in organizations (Di Fabio, 2017).

## Psychology of Sustainability and Sustainable Development

The psychology of sustainability and sustainable development (Di Fabio, 2016a, 2017) sees sustainability not only in terms of the ecological and socio-economic environment (Brundtland Report, 1987) but also in terms of improving the quality of life of every human being.

More specifically, the word “sustainable” refers etymologically to something that can be sustained for a period of time. It refers also to something that can be supported, tolerated, or confirmed over time, and that can be stated with certainty. It concerns building on the present in such a way as not to put the future at risk. In politics, technology, the economy, and the ecology, sustainability is about balancing current aims with future aims without jeopardizing the latter (Di Fabio, 2016a; Di Fabio and Maree, 2016).

Traditionally, sustainable development (Brundtland Report, 1987; Harris, 2003) was based on the three “Es” of economy, equity, ecology, and highlighting the right of present as well as future generations to enjoy the environment and natural resources. Psychologically, sustainability is viewed not only in terms of the ecological and social environment but also in terms of promoting the well-being of all people (Di Fabio, 2016a). While the traditional definition of sustainability focuses on avoiding (exploitation, depletion, and irreversible alteration), the new definition focuses on promoting (enrichment, growth, and flexible change) (Di Fabio, 2016a).

According to the traditional point of view, a product is sustainable if it uses increasingly smaller amounts of material; if it is based on renewable and non-polluting processes and materials; if it is not toxic; and if it is easy to maintain, process, dismantle, demolish, dispose of, and recycle (Di Fabio, 2016a). According to the new point of view, the construction and managing of a sustainable project is based not only on using increasingly smaller amounts of resources but also on regenerating resources (Di Fabio, 2016a). A sustainable project is thus accessible, de-constructible, and recoverable and comprises oxygenating processes aimed at promoting individual and organizational well-being (Di Fabio, 2016a; Di Fabio and Maree, 2016). A sustainable project proposes what does not yet exist; it changes what exists according to new goals to achieve new results; and it transfers knowledge and solutions to meet new challenges (Vygotskij, 1934; Di Fabio, 2002, 2014c, 2016a).

Reflexivity processes call for meaning (Guichard, 2004, 2010; Maree, 2013; Di Fabio, 2014c, 2016a) and are important to ensure sustainability in 21st century organizations (Di Fabio, 2014c, 2017). A meta-centric reflexivity perspective for sustainability (Di Fabio, 2016a) has been devised to help these processes. In details, Di Fabio (2016a) argues that the sustainability of a project from a psychological point of view involves vertical and horizontal axes of reflexivity that can be articulated in terms of micro-dimensions and macro-dimensions. The vertical axis involves the idea of “where I come from,” establishes awareness of “where I am,” and proceeds to “where I will go.” The horizontal axis, conversely, concerns the transition from an egocentric, self-centered position to a new altruistic meta-centric position focused on the promotion of mutual gain, namely gain for others and gain for the self on the one hand, and connectedness focused on reflexivity (from the micro- to the macro-level) on the other hand. Here, the sustainability of a project is based on the identification of the zone of proximal sustainable development for the individual (Vygotskij, 1934; Di Fabio, 2002, 2014c, 2016a). Regarding the horizontal axis of the sustainability of a project, the transition takes place from a micro-level to a macro-level in the relationship between people and their world. The meta-centric reflexivity approach to sustainability (Di Fabio, 2016a) is a further innovation in the psychology of sustainability and sustainable development.

The sustainability of a project is key to well-being from a primary prevention point of view (Di Fabio, 2016a). Developing awareness of this fact is particularly useful not only in relation to the individual but also in relation to the group and the organization. Together with this new awareness is the need to find a balance between “me,” “we,” “organization,” “people,” and “the world.” A meta-centric reflexivity approach to sustainability is therefore essential for well-being in the highly fluid organizations of the 21st century (Guichard, 2013; Di Fabio, 2016a).

A reflective grid for the sustainability of personal projects (adapted by Blanché, 1957) has been developed (Di Fabio, 2016a), but this grid can also be used to improve the sustainability of group and organization projects. The grid permits reflection on the following points: sustainabilityness, no sustainabilityness; crisis of sustainabilityness, no crisis of sustainabilityness; some sustainabilityness, some crisis of sustainabilityness; neither sustainabilityness, nor crisis of sustainabilityness (Di Fabio, 2016a). The use of the grid in organizations at the various levels (individual, group, and organization) can also enhance awareness of the real areas of strength and sustainable development.

## Psychology of Sustainability and Sustainable Development for Well-Being in Organizations

The psychology of sustainability and sustainable development (Di Fabio, 2016a, 2017) reinforces the primary prevention approach (Di Fabio and Kenny, 2015, 2016) and fosters well-being in organizations at all the various levels, starting from the worker and going on to the group, to the organization, and to organizational and inter-organizational processes.

In this approach, the meaningfulness of the project plays a new and vital role in its real sustainability (Di Fabio, 2016a), whether it is a work-life project, a group project, an organizational project, or an inter-organizational project. Projects are more sustainable if they are characterized by coherence, direction, significance, and belonging (Schnell et al., 2013; Di Fabio, 2016a). Here, it is important to stress the passage from the motivational paradigm to the meaning paradigm (Di Fabio and Blustein, 2016). The motivational paradigm concerns intrinsic motivation (doing a job to gain satisfaction), extrinsic motivation (doing a job for reward or to avoid a punishment), and lack of motivation (lack of awareness of the link between behavior and consequences) (Tremblay et al., 2009; Deci and Ryan, 2010). The meaning paradigm concerns understanding how people can establish meaningful lives and meaningful work experiences in the midst of numerous challenges, transitions, and changes. The sustainability of a life-work project needs to be anchored to a meaningful life-work construction (Di Fabio and Blustein, 2016) so that the project can be truly viable thereby enhancing people’s involvement and increasing the chances of success.

Regarding sustainable life-work projects, it is important to consider job satisfaction, job crafting, job design, and job redesign very carefully. Job satisfaction refers to the positive emotional state arising from the evaluation of employees’ job experience (Locke, 1976) in terms of their relationships with colleagues and supervisors, job rewards in terms of monetary compensation and promotion, and quality of working conditions (Spector, 1997, 2008; Drydakis, 2012, 2015). Job crafting refers to how employees modify the form, scope, and extent of work activities according to their own skills, needs, and preferences (Wrzesniewski and Dutton, 2001) thereby enhancing their well-being in terms of, for example, job satisfaction (Hakanen et al., 2017) and work engagement (Demerouti, 2014). The above concepts of job satisfaction and job crafting are linked also to job design as a process that determines how jobs, tasks, and roles are structured, implemented, and changed, as well as their influence on individuals, groups, and organizational outcomes (Grant and Parker, 2009). Job redesign refers to the modification of the jobs, the tasks, and the conditions of work of employees (Tims and Bakker, 2010) with the aim of improving their work motivation and performance (Le Blanc et al., 2017).

A primary prevention approach aimed at ensuring well-being is the key for sustainability, growth, and success for workers, groups, and organizations (Di Fabio, 2016a, 2017).

Greater organizational awareness of psychologically sustainable development is needed to facilitate positive narratives at the personal, teamwork, and organizational level. Organizational narratives, which are often complicated and negative, can be transformed through processes of reflexivity that can generate meaning, hope, new possibilities, success, and sustainable development (Di Fabio, 2016a, 2017).

According to the psychology of working (Blustein, 2006), work can fulfill different needs such as power needs, relationship needs, and self-determination needs. Relationships can thus be considered a fundamental aspect of working. The relational theory of working (Blustein, 2011) holds that work is an inherently relational act as relationships influence and shape every decision, experience, and the interaction of individuals in the world of work. Work meets not only the need for survival but also the need for social connection. This underlines the importance of relationships that are built in the reality of each single moment and each day of working life in organizations. Relationships consist also of the meanings constructed and shared in organizational contexts.

The psychology of sustainability and sustainable development thus calls for managerial styles and leadership that recognize and respect the importance of relationships in organizational contexts for the well-being of workers. Leadership includes directing the actions of an organizational group to reach a goal (House et al., 1999; Boyatzis, 2006), whereas management involves mainly organizing and coordinating projects and making projections (Michael et al., 2002; Renko et al., 2015). The literature covers different leadership styles (Eagly et al., 2003) including new leadership styles such as sustainable leadership (Hargreaves et al., 2003; Hargreaves and Fink, 2004), servant leadership (Ehrhart, 2004), authentic leadership (Avolio et al., 2009), ethical leadership (Gallagher and Tschudin, 2010), mindful leadership (George, 2012; Herold, 2013), benevolent leadership (Wang and Cheng, 2010), and decent leadership (Di Fabio, in press). Sustainable leadership refers to the shared responsibility to preserve human and economic resources as far as possible and to avoid social and environmental degradation (Hargreaves et al., 2003; Hargreaves and Fink, 2004). Servant leadership refers to putting followers’ growth and interests above the aims of the organization or of the leaders (Ehrhart, 2004). Authentic leadership refers to focusing virtuously on followers’ resources and strengths rather than their weaknesses (Avolio et al., 2009). Ethical leadership refers to striving after ethical goals and the empowerment of followers (Gallagher and Tschudin, 2010). Mindful leadership refers to concentrating on the present moment and recognizing and controlling feelings and emotions, particularly in stressful situations. Mindful leadership refers to being aware of the presence of followers and of leaders’ influence on them (George, 2012; Herold, 2013). Benevolent leadership refers to focusing on followers’ welfare at work as well as their personal lives, including their family members (Wang and Cheng, 2010). Finally, decent leadership involves the above concepts of leadership (sustainable, servant, authentic, ethical, mindful, and benevolent) as well as the management of diverse resources in an organization (Di Fabio, in press).

A new organizational sensibility is required to manage, promote, and ensure sustainable development in “liquid” organizations in uncertain and ever-changing environments. A managerial approach and new styles of leadership that show awareness of the importance of relationships and of constructing positive narratives in organizational contexts is the key to mobilizing energy, coping with challenges, and promoting sustainable development and the well-being of people in organizations.

## Conclusion

The psychology of sustainability and sustainable development calls for new awareness of the need to achieve sustainable well-being from a primary prevention point of view. This involves designing and constructing organizational development and well-being through the promotion of relationships and positive narratives (Di Fabio, 2017) in organizational contexts in everyday life.

A lot of research and interventions based on positive psychology are available to improve leadership skills and human resources management for managers in 21st century organizations. For example, from emotional intelligence (Petrides and Furnham, 2001; Di Fabio and Saklofske, 2014a,b; Di Fabio et al., 2016b) to empathy (Davis, 1980; Di Fabio, 2014b; Di Fabio and Bucci, 2016), compassion (Martins et al., 2013), and self-compassion (Neff, 2003); from positive capital (Luthans et al., 2007) to intrapreneurial self-capital (Di Fabio, 2014a) as a core of individual intrapreneurial resources to deal with frequent changes and transitions and to turn constraints into resources, to acceptance of change (Di Fabio and Gori, 2016b) as positive for a person’s well-being from positive relational management (Di Fabio, 2016b) to workplace relational civility (Di Fabio and Gori, 2016a) as a relational style characterized by respect and concern for the self and others, and by interpersonal sensitivity (relational decency, relational culture, and relational readiness), to decent leadership (Di Fabio, in press); from reflexivity in its dimensions of clarity/projectuality, authenticity, and acquiescence (Di Fabio, 2015, 2016a) to the meaning of work and life (Bernaud, 2015; Di Fabio and Blustein, 2016). New research and intervention are needed to better explore and understand these issues. Furthermore future directions can also consider that healthy societies and healthy organizations can be enhanced by focusing on the well-being of individuals, groups, and organizations in a culturally diverse world. This can best be done from a cross-cultural point of view and on the basis of the psychology of sustainability and sustainable development (Di Fabio, 2017). Cross-level interactions are needed that combine individual approaches and collective approaches, and short-term and long-term objectives and results (Hofmann and Tetrick, 2003), thereby providing for timeous interventions from a primary prevention perspective (Peiró, 2008; Tetrick and Peiró, 2012), reducing personal and contextual threats, and increasing health and well-being (Di Fabio and Kenny, 2015, 2016).

Challenges are essentially opportunities. The psychology of sustainability and sustainable development can be seen as an adaptive response to the need to develop well-being in organizations that have to cope with the challenging and unpredictable environments of the 21^st^ century.

## Author Contributions

AD ideated the structure, analyzed the literature, and wrote the manuscript.

## Conflict of Interest Statement

The author declares that the research was conducted in the absence of any commercial or financial relationships that could be construed as a potential conflict of interest. The reviewer CG-A, and handling Editor declared their shared affiliation, and the handling Editor states that the process nevertheless met the standards of a fair and objective review.
